# Evaluating the Effect of Flame for the Determination of Carbohydrate, Protein, and Dietary Fiber in Nepali Food Dhindo-Novel Food for Diabetic

**DOI:** 10.1155/2020/8832151

**Published:** 2020-09-08

**Authors:** Hemraj Sharma, Puja Pudasaini, Saraswati Dhungana, Manisha Pokharel, Punam Subedi, Bedraj Sharma

**Affiliations:** ^1^Department of Pharmacy, Shree Medical and Technical College, Bharatpur, Chitwan, Nepal; ^2^Department of Food technology, Nagarik College, Gaindakot, Chitwan, Nepal

## Abstract

Dhindo is a thick pasty Nepalese porridge prepared by cooking grounded, millet, or cornmeal flour. It is a staple meal in various parts of Nepal, especially in hilly areas. It is prepared by gradually adding flour to boiling water while stirring. Due to its soft pasty nature, it can be eaten by any age of people; in particular, it is good for old age people. As majority of the world population has been suffering from diabetes and dhindo being believed to have less carbohydrate content, hence, this study can serve as great nutritional value to a large number of diabetic patients. The present study was undertaken to determine nutrient contents from the novel food dhindo, which is prepared from different flours (maize, wheat, millet, and buckwheat) and to compare its nutrients with rice. Dhindo and rice were prepared and analyzed for total carbohydrate, reducing sugar, protein, and fiber. Here, we compared all the nutrients of dhindo and rice as well as determined the effect of heat on the contents of dhindo and rice. Dhindo and rice were cooked, and all the samples were evaluated for the nutritional contents. Dietary fiber was determined from the gravimetric method. The millet form of dhindo contained a high amount of dietary fiber, which was found to be 0.835 gm by the firewood method and 0.82 gm by LPG gas. Total carbohydrate, reducing sugar, and protein were determined from the UV visible spectrophotometer. Rice contained a high amount of total and reducing sugar and was found to be 31.8 mg/3 gm and 30.03 mg/3 gm by LPG and firewood, respectively, for total carbohydrates and 0.218 mg/3 gm and 0.214 mg/3 gm by LPG and firewood, respectively, for reducing sugars. The protein was found to be maximum in the buckwheat form of dhindo, which was 15.892 mg/1 gm and 15.375 mg/1 gm by LPG and firewood, respectively. From this study, we can conclude that consuming dhindo would be advantageous for a diabetic patient than rice.

## 1. Introduction

Dhindo is the Nepali national food that is made from flour of different grains like buckwheat, millet, wheat, and maize. It is a typical Nepali food consumed by Nepalese people from ancient times. It is rich in nutrients and is traditionally prepared from ground buckwheat, millet, and wheat, or corn meal is common as well. Theoretically, any flour can be used to make dhindo, as it is essentially a simple mixture of hot water and ground grain. Buckwheat (Fagopyrum esculentum Moench) is an important food stuff in certain areas of the world. Besides providing protein and energy, buckwheat is a treasured source of minerals like zinc, dietary copper, and manganese [1, 2]. Millet (Panicum miliaceum) is a cereal with high protein content, low fat, very high fiber, high amount of lecithin, rich in vitamin B, niacin, folic acid, calcium, iron, zinc, etc. Beside of human consumption, it has been used as livestock and bird feed [3]. Wheat (Triticum) is a common crop consumed almost in all parts of world and is rich in protein, carbohydrate, iron, zinc, selenium, and manganese [4]. Corn (Zea mays) is a popular starchy food and cereal grain, eaten all over the world for centuries, and is rich in carbohydrate, protein, potassium, calcium, sulphur, etc. [5]. Proteins are extremely complex nitrogenous organic compounds which are polymers of amino acid and possess high molecular weight and are estimated by a colorimeter and spectrophotometer, through a chromatography method (HPLC, IEC) using various quantification methods such as BCA (bicinchoninic acid), Bradford method, Folin-Lowry method, Kjeldahl method, and Ultraviolet absorption method [6]. The right amount of protein helps to manage diabetes in a few ways. First of all, it will help take the edge of all hunger and can speed up our blood sugar level and pairing protein-rich foods with carbohydrates slows the rise in blood sugar. Carbohydrate is generally the compounds of carbon, hydrogen, and oxygen, but higher carbohydrates have also been found to possess nitrogen and sulphur in their structure [5] and may be present as isolated molecules, or they may be physically associated or chemically bound to other molecules. Along with the nutritional aspects, they also contribute to the sweetness, appearance, and textural characteristics of many foods. Carbohydrates can be estimated by the colorimetric method (phenol-sulphuric acid method), chromatography technique (HPLC, GC, and TLC), electrophoretic method, gravimetric method (Munson and walker method), and spectrophotometer [7, 8]. Fibers may be defined as any hair-like raw material directly obtainable from an animal, vegetables, or mineral source, and their content in food can be determined either gravimetrically by weighing the mass of an insoluble fiber fraction isolated from a sample or chemically by breaking down fiber into constituent monosaccharide and measuring their concentration [9], and it helps to control blood sugar levels. It slows the absorption of sugar and helps improve blood sugar levels. A healthy diet that includes insoluble fiber may also reduce the risk of developing type 2 diabetes. Soluble fiber helps to lower total blood cholesterol levels by lowering low-density lipoprotein or bad cholesterol levels [10]. Temperature plays an important role in the estimation of carbohydrate, protein, and dietary fiber. Protein denaturation occurs in high as well as low temperatures. The heat disrupts hydrogen bond and nonpolar hydrophobic interactions, and with the decreasing temperature, the free energy cost for hydrophobic effect or unfavorable interaction of nonpolar residues with water decreases thus increasing their hydration and denaturation occurs (cold denatured system) [11]. Similarly for dietary fiber, heat loses the water-soluble fiber and significantly more water-insoluble dietary fiber as compared to raw samples [12]. Carbohydrates tend to react or decompose upon heating. This is because of a reactive group such as carbonyls that find electron-poor regions of other molecules to interact with, such as amines. Also, hydrogen bonds between neighboring molecules can be strong, in aggregate, as internal bonds within the molecule [11]. Till now, there is a lack of study for the exact amount of essential constituents in Nepali food dhindo, which is generally consumed by people. It is believed to contain less carbohydrate than normal rice or other carbohydrate-containing food, but till now, there is no evidence for it. Generally, carbohydrates, proteins, and fibers are the most important chemical constituents that directly affect the health of an individual. As flame may cause alteration in amount, the study on the effect of low flame intensity by using firewood (ancient method) and high flame intensity by using LPG gas (modern method) on the content of food is not studied, to date. The study on the dhindo is done for the estimation of the different constituents present in the food. Dhindo is cooked in high flame and low flame, and the content of food such as carbohydrate, protein, and fiber was estimated. The effect of heat on the content of foodstuffs such as protein, carbohydrate, and fiber was determined. The comparison was done between dhindo and rice to find out which ones contain less carbohydrate as well as reducing sugar and the protein, as well as fiber contents.

## 2. Materials and Methods

### 2.1. Materials and Reagents

The raw materials (maize flour, wheat flour, millet flour, buckwheat flour, and rice) were obtained from local sources. Sulphuric acid, hydrochloric acid, sodium hydroxide pellets, diethyl ether, DNSA (3, 5, dinitrosalicyclic acid), protein standard (1 mg/ml), Folin-Ciocalteu's phenol reagent, and biuret reagent were provided by Shree Medical and Technical College.

### 2.2. Study Design

It was an experimental study as the samples were prepared, and the estimation of protein, carbohydrate, and dietary fiber was done and compared.

### 2.3. General Procedure of Cooking Dhindo

Dhindo was prepared by heating water in pan till it boiled, and flour was added as required and triturated continuously until it became solid and triturated for some time to make uniform appearance.

### 2.4. Study Methods

Since various analytical procedures were conducted in UV visible in spectrophotometer, the linearity curve was drawn to conduct the relationship between concentration and absorbance. The estimation of protein, carbohydrate, and dietary fiber of sample was done by comparing with the standard linearity curve. Finally, the amount of contents in samples cooked in LPG gas and firewood was compared.

### 2.5. Methods

#### 2.5.1. Estimation of Dietary Fiber

The determination of dietary fiber was carried out by gravimetry using Sadasivam and Manickam's method as a reference ^[^13^]^. Two grams of dried food was extracted with ether followed by treatment with sulphuric acid and sodium hydroxide. Finally, the mass was filtered, washed, dried, and weighed.

#### 2.5.2. Estimation of Carbohydrate

For total carbohydrate and reducing Sugar, the determination was conducted as per PerkinElmer (2015-2016) [14] by extracting the sample with distilled water. The concept used was to change the colorless sample to color complex by the chemical derivatizing method; for such, the Dinitrosalicylic acid solution was used as a derivatizing agent. To estimate the total carbohydrate, the samples were analyzed at 580 nm and for reducing sugar at 600 nm.

#### 2.5.3. Estimation for Protein

The experiment was conducted as per PerkinElmer (2015-2016) [15]. The sample was extracted with saline solution, and the extracted protein from Dhindo (colorless) was changed to color complex by adding biuret and Folin-Ciocalteu's phenol reagent, and absorbance was measured at 725 nm.

## 3. Results

### 3.1. Calibration Curve

**Figure 1:**
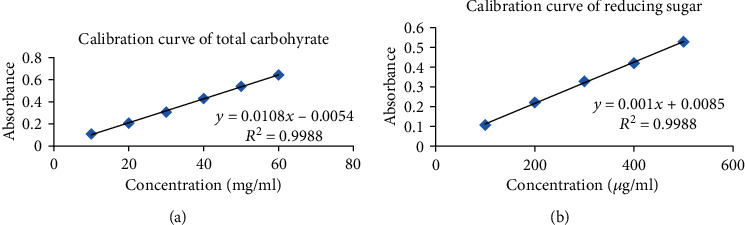
(a) Standard calibration curve of total carbohydrate. (b) Standard calibration curve of reducing sugar.

The standard calibration curve of the total carbohydrate for absorbance versus concentration (10, 20, 30, 40, and 50 mg/ml) (Figure 1(a)) and absorbance was measured at 580 nm. Similarly, the standard calibration curve of reducing sugar for absorbance versus concentration (100, 200, 300, 400, 500 *μ*g/ml) (Figure 1(b)) and absorbance was taken at 600 nm using UV visible spectrophotometer; then, the calibration curve was plotted by taking absorbance on the *y*-axis and concentration on *x*-axis. The correlation coefficient was calculated from the graph.

**Figure 2:**
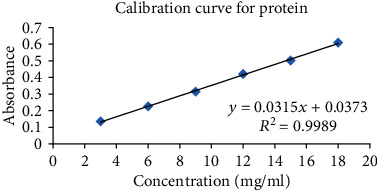
Standard calibration curve of protein.

The standard calibration curve of protein for absorbance versus concentration (3, 6, 9, 12, 15, and 18 mg/ml) was constructed, (Figure 2) and absorbance at 725 nm was measured using a UV-visible spectrophotometer. The correlation coefficient was calculated from the graph.

#### 3.1.1. Pilot Study

A pilot study was performed to determine the exact amount of ingredients for analysis. Dhindo was prepared out by taking various weights of ingredients; finally, uniform dense slurred mass was obtained by taking 40 grams of maize and wheat and 50 grams of millet and buckwheat. 150 ml of water was taken as vehicle for preparation. Hence, this formulation was used for the analysis.

#### 3.1.2. For Dietary Fiber

Dhindo (maize, wheat, millet, and buckwheat) and rice, cooked in LPG gas and firewood, were analyzed for dietary fiber (Table 1).

The experimental values of sample cooked in LPG gas and firewood were compared statistically by the Student *t*-test showing no significant difference (*p* = 0.91, i.e., *p* > 0.05). As shown in Table 1, the highest fiber content was obtained in millet followed by buckwheat, maize, and wheat, and rice is found to have lowest fiber content. The dietary fiber cooked in firewood is slightly greater than the samples cooked in LPG gas.

### 3.2. Calculation

For the calculation of fiber content following formula was used.

(1) $$\begin{array}{c} \%\text{crude fiber in ground sample}=\left(\text{loss in weight on ignition }\left(w_{2}-w_{1}\right)-\left(w_{3}-w_{1}\right)/\text{weight of sample}\right)\times 100, \end{array}$$

where *w*_1_ = weight of empty filter paper, *w*_2_ = weight of residue after filtration, and *w*_3_ = weight of residue after drying.

#### 3.2.1. For Total Carbohydrate

Dhindo (maize, wheat, millet, and buckwheat) and rice, cooked in LPG gas and firewood, were analyzed for total carbohydrate (Table 2).

The experimental values of sample cooked in LPG gas and firewood were compared statistically by the Student *t*-test showing no significant difference (*p* = 0.71, i.e., *p* > 0.05). As shown in Table 2, the highest total carbohydrate content was found in rice followed by wheat, maize, millet, and buckwheat.

#### 3.2.2. For Reducing Sugar

Dhindo (maize, wheat, millet, and buckwheat) and Rice, cooked in LPG gas and firewood, were analyzed for reducing sugar (Table 3).

The experimental values of sample cooked in LPG gas and firewood were compared statistically by the Student *t*-test showing no significant difference (*p* = 0.86, i.e., *p* > 0.05). As shown in Table 3, the highest reducing sugar content is of rice followed by wheat, maize, buckwheat, and millet.

#### 3.2.3. For Protein

Dhindo (maize, wheat, millet, and buckwheat) and rice, cooked in LPG gas and firewood, were analyzed for proteins (Table 4).

The experimental values of sample cooked in LPG gas and firewood were compared statistically by the Student *t*-test showing no significant difference (*p* = 0.88, i.e., *p* > 0.05). As shown in Table 4, the highest protein content was found in buckwheat, wheat, maize, millet, and rice.

## 4. Discussion

The purpose of the study was to evaluate and compare the total carbohydrate content, Reducing sugar, protein and dietary fiber of the dhindo (maize, wheat, millet, and buckwheat) and rice cooked in the LPG gas and firewood. The total carbohydrate content as well as reducing sugar content was found to be higher in rice followed by wheat, maize, buckwheat, and millet. Dietary fiber was found to be more in millet followed by buckwheat, maize, and wheat and lower in rice. Protein content was found to be abundant in buckwheat followed by wheat, maize, millet, and rice. As we compared the total carbohydrate content, reducing sugar, and protein and dietary fiber contents of the samples cooked in LPG gas and firewood, it was found that the dietary fiber of samples cooked in firewood is slightly greater than the samples cooked in LPG gas and total carbohydrate content, reducing sugar, and protein content of samples cooked in LPG gas is slightly greater than the samples cooked in Firewood.

A.D.A.M. Medical Encyclopedia (2019) mentioned that 1 bowl of rice consists of 69 gm carbohydrate, protein 6 gm. Similarly, 1 bowl of maize consists of 25 gm of carbohydrate; 3 gm of protein, and wheat contain 2 1gm of carbohydrate, 4 gm of protein. Buckwheat contains 71 gm of carbohydrate, 13 gm of protein. Similarly, millet contains 41 gm carbohydrates and 6 gm of protein [16]. As we found that the total carbohydrate and reducing sugar in rice is lowest in the buckwheat and millet as compared to others samples as well as fiber contents are also found to be highest in these two samples, it can be better for consumption for the diabetic patients as compared to other samples. The study discusses the preparation of various samples of dhindo (maize, wheat, millet, and buckwheat) and rice in LPG gas as well as firewood and involves the evaluation of the contents of the various samples. The fiber contents of samples cooked in firewood was found to be slightly greater than that of the samples cooked in LPG gas. Total carbohydrate, reducing sugar, and protein of samples cooked in LPG gas was found to be greater than that of the samples cooked in firewood. Total carbohydrate as well as reducing sugar was found to be highest in rice and lowest in buckwheat. Fiber content was found to be highest in millet followed by buckwheat, maize, wheat, millet, and rice. So we can conclude that for the diabetic patients, dhindo of millet and buckwheat can be a better consumption as compared to the consumption of rice.

## 5. Conclusion

By using the gravimetric method, dietary fiber was determined and the UV visible spectrophotometry method was used to determine total carbohydrate, reducing sugar, and protein. From the study, we can suggest that the people suffering from diabetics are advised to have dhindo rather than rice. Among different flours, dhindo of buckwheat and millet was found to be better as these contain high dietary fiber as well as low total carbohydrate and reducing sugar. As dietary fiber was found to be higher in sample cooked in firewood as well as total carbohydrate and reducing sugar were found to be less in it, so dhindo cooked in firewood was found to be better for consumption.

## Figures and Tables

**Table 1:** Dietary fiber in sample cooked in LPG gas and firewood.

Raw materials	Dietary fiber in sample cooked in LPG gas (gm) mean ± S.D (*n* = 3)	Dietary fiber in sample cooked in firewood (gm) mean ± S.D (*n* = 3)
Maize	0.75167 ± 0.0246	0.7634 ± 0.038
Wheat	0.63 ± 0.0589	0.643 ± 0.005
Millet	0.82 ± 0.0752	0.835 ± 0.032
Buckwheat	0.81167 ± 0.05	0.8267 ± 0.040
Rice	0.2433 ± 0.043	0.2833 ± 0.052

**Table 2:** Total carbohydrate content in sample cooked in LPG gas and firewood.

Raw materials	Total carbohydrate in sample cooked in LPG gas (mg/3 gm) mean ± S.D (*n* = 3)	Total carbohydrate in sample cooked in firewood (mg/3 gm) mean ± S.D (*n* = 3)
Maize	14.1 ± 1.708	11.1 ± 1.47
Wheat	18.9 ± 1.058	16.867 ± 1.60
Millet	10.96 ± 0.2516	9.83 ± 1.18
Buckwheat	11.96 ± 1.553	9.43 ± 1.715
Rice	31.8 ± 0.754	30.03 ± 1.70

**Table 3:** Reducing sugar content in sample cooked in LPG gas and firewood.

Raw materials	Reducing sugar in sample cooked in LPG gas(mg/3 gm) mean ± S.D (*n* = 3)	Reducing sugar in sample cooked in firewood(mg/3 gm) mean ± S.D (*n* = 3)
Maize	0.1293 ± 0.0359	0.113 ± 0.0528
Wheat	0.131 ± 0.0208	0.129 ± 0.01
Millet	0.0356 ± 0.0111	0.0233 ± 0.005
Buckwheat	0.059 ± 0.007	0.0463 ± 0.0106
Rice	0.218 ± 0.0295	0.214 ± 0.034

**Table 4:** Protein content in sample cooked in LPG gas and firewood.

Raw materials	Protein in sample cooked in LPG gas(mg/1 gm) mean ± S.D (*n* = 3)	Protein in sample cooked in firewood(mg/1 gm) mean ± S.D (*n* = 3)
Maize	8.429 ± 1.315	7.279 ± 0.512
Wheat	13.139 ± 0.6619	12.967 ± 0.4866
Millet	6.621 ± 0.589	6.182 ± 0.3551
Buckwheat	15.892 ± 0.9135	15.375 ± 0.4388
Rice	1.536 ± 0.3696	1.25 ± 0.225

## Data Availability

The data used to support this study are available from the corresponding author upon request.
